# Molecular Logic as a Means to Assess Therapeutic Antidotes

**DOI:** 10.3389/fchem.2019.00243

**Published:** 2019-04-16

**Authors:** Linor Unger-Angel, Leila Motiei, David Margulies

**Affiliations:** Department of Organic Chemistry, Weizmann Institute of Science, Rehovot, Israel

**Keywords:** molecular logic gates, fluorescent molecular probes, supramolecular chemistry, enzyme inhibitors, thiazole orange-based protein identifiers (TOPIs), glutathione s-transferase (GST), cyclodextrin host, adamantyl guest

## Abstract

An emerging direction in the area of molecular logic and computation is developing molecular-scale devices that can operate in complex biological environments, such as within living cells, which are beyond the reach of conventional electronic devices. Herein we demonstrate, at the proof-of-principle level, how concepts applied in the field of molecular logic gates can be used to convert a simple fluorescent switch (YES gate), which lights up in the presence of glutathione s-transferase (GST), into a medicinally relevant INHIBIT gate that responds to both GST and beta-cyclodextrin (β-CD) as input signals. We show that the optical responses generated by this device indicate the ability to use it as an enzyme inhibitor, and more importantly, the ability to use β-CD as an “antidote” that prevents GST inhibition. The relevance of this system to biomedical applications is demonstrated by using the INHIBIT gate and β-CD to regulate the growth of breast cancer cells, highlighting the possibility of applying supramolecular inputs, commonly used to control the fluorescence of molecular logic gates, as antidotes that reverse the toxic effect of chemotherapy agents. We also show that the effect of β-CD can be prevented by introducing 1-adamantanecarboxylic acid (Ad-COOH) as an additional input signal, indicating the potential of obtaining precise, temporal control over enzyme activity and anticancer drug function.

## Introduction

Molecular logic gates constitute a wide range of molecular switches that respond to diverse input signals according to the rules of Boolean logic. Since the inception of the first molecular AND logic gate (de Silva et al., 1993), a wide range of “digital” optical switches (de Ruiter and van der Boom, 2011; de Silva and Uchiyama, 2011; Magri and Mallia, 2013; Ling et al., 2015; Akkaya et al., 2017; Katz, 2017; Erbas-Cakmak et al., 2018; Pilarczyk et al., 2018) and related pattern-generating devices (Rout et al., 2012, 2013, 2014; Sarkar et al., 2016; Hatai et al., 2017; Lustgarten et al., 2017; Pode et al., 2017) have been developed and used for various applications, including information processing (de Ruiter and van der Boom, 2011; de Silva and Uchiyama, 2011; Ling et al., 2015; Akkaya et al., 2017; Katz, 2017; Erbas-Cakmak et al., 2018; Pilarczyk et al., 2018), user identification (Rout et al., 2013; Lustgarten et al., 2017; Andréasson and Pischel, 2018), cryptography (Sarkar et al., 2016; Lustgarten et al., 2017), and sensing (Ling et al., 2015; Wu et al., 2017; Erbas-Cakmak et al., 2018; Magri, 2018). These input-dependent systems were initially considered as potential alternatives to conventional transistors and circuits (de Silva et al., 1993). However, as the area of molecular computing progressed, and as the challenges associated with building molecule-based circuits revealed, this field has undergone a paradigm shift. It is now believed that although molecular logic gates may not be able to compete with the speed and power of silicon, a key advantage of using such systems lies in their molecular scale, which enables them to target and sense small biomolecules (e.g., proteins) and to operate in living cells and tissues, where silicon-based devices cannot access (de Silva, 2013). Examples of such systems include molecular logic gate-based photodynamic therapy agents designed to be less harmful to neighboring healthy tissue (Erbas-Cakmak and Akkaya, 2013; Erbas-Cakmak et al., 2013, 2015; Turan et al., 2018), as well as “digitally activated” drug models (Amir et al., 2005; Wang et al., 2010; You et al., 2015; Pode et al., 2017) that release an active pharmaceutical in response to combinations of biomarkers. We have recently shown that “digital” control over prodrug activation can also be achieved by controlling communication between proteins, rather than modifying the prodrug itself (Peri-Naor et al., 2015a,b). The “smart” therapeutic models mentioned before (Amir et al., 2005; Wang et al., 2010; de Silva, 2013; Erbas-Cakmak and Akkaya, 2013; Erbas-Cakmak et al., 2013, 2015; Peri-Naor et al., 2015a,b; You et al., 2015; Pode et al., 2017; Turan et al., 2018) demonstrate how concepts developed in the area of molecular logic may ultimately lead to the generation of highly selective anti-cancer drugs. The latter may not only minimize the severe side effects of current chemotherapies—these drugs may also prevent the increasing incidence of patients' death when inappropriate doses are mistakenly used.

An alternative approach to preventing the lethal effects of improper chemotherapy treatment has recently emerged. Rather than developing more selective drugs, this approach relies on “chemotherapy antidotes” that compete with the binding of chemotherapy agents to their protein targets and consequently, arrest their fatal effects. Vistogard (uridine triacetate), for example, is an antidote that has recently been approved for treating life-threatening toxicity patients subjected to an overdose of the chemotherapy medication capecitabine (5-fluorouracil or 5-FU) (Cada et al., 2016). Reversing the effect of therapeutic agents can also be achieved by using synthetic agents that interact with the synthetic agonist/antagonist themselves, rather than with their biological target. Such systems could allosterically change the structure of an inhibitor (Taylor et al., 2009; Zhou et al., 2017; Mukherjee et al., 2018) or encapsulate it via supramolecular host-guest interactions (Bom et al., 2002). We have recently developed an allosteric inhibitor that reversibly controls the activity of glutathione s-transferase (GST), and we have used Boolean logic to analyze the inhibitor's function (Peri-Naor et al., 2015a). In parallel, we developed “turn-on” fluorescent molecular probes (i.e., YES gates), termed thiazole-orange-based protein identifier (TOPIs), which detect GST by generating a light output signal. Herein, we show that digital analysis of the response of one of these probes (TOPI-4) to GST and an additional chemical input (β-CD) converts this probe from a simple fluorescent YES gate into an INHIBIT gate. Previous studies have demonstrated the ability to obtain molecular INHIBIT gates that respond to supramolecular hosts as inputs (de Silva et al., 1999; Pischel et al., 2010). In this study, we show that such digital optical responses can be used to monitor GST binding interactions and, as a result, indicate whether β-CD could prevent the molecular logic gate from binding and inhibiting GST. Bearing in mind the wide interest in creating chemotherapy antidotes (Cada et al., 2016), we demonstrate, at the proof-of-principle level, how the toxic effect of this inhibitor on cancer cells can be regulated using external input signals.

## Materials and Methods

All reagents and solvents were obtained from commercial suppliers and used without further purification. Recombinant human GST P1-1 was obtained from the Israel Structural Proteomics Center (Weizmann Institute of Science, Rehovot, Israel). 1-Adamantanecarboxylic acid and β- cyclodextrin were purchased from Sigma-Aldrich. Enzymatic assays were carried out using a BioTek synergy H4 hybrid multiwell plate reader in clear flat-bottom polystyrene 384-well microplates (Corning). Fluorescence measurements were carried out using a BioTek synergy H4 hybrid multiwell plate reader in clear and black flat-bottom polystyrene NBS 384-well microplates (Corning).

### GST Activity Measurements

The GST activity was measured spectrophotometrically in aqueous phosphate buffer (5 mM, pH 6.5) using chloro-2,4-dinitrobenzene (CDNB) and Glutathione (GSH) as substrates. The concentrations of GST-P1-1, GSH, and CDNB were 20 nM, 350 μM, and 700 μM, respectively. In a typical experiment, TOPI-4 and various concentrations of β-CD were incubated in microplate wells and then GST-P1-1, GSH, and CDNB were subsequently added. The formation of S-(2,4-dinitrophenyl)-glutathione was monitored using a microplate reader at λ = 340 nm.

### Controlling GST Activity With β-CD and Ad-COOH

TOPI-4 (500 nM) was incubated with various concentrations of β-CD and Ad-COOH for 10 min and then GST-P1 (20 nM) was added. Finally, GSH (350 μM) and CDNB (720 μM) were added and the initial kinetic velocities (V_0_) were measured. Data are expressed as mean values ± std. deviation of triplicate measurements.

### Controlling the Fluorescence Response With β-CD and Ad-COOH

TOPI-4 (100 nM) was incubated with various concentrations of β-CD and Ad-COOH for 10 min and then GST-P1 (90 nM) was added. The fluorescence measurements were recorded after 30 min of incubation in aqueous phosphate buffer (5 mM, pH = 6.5) using a microplate reader and an excitation wavelength of 500 nm. Data are expressed as mean values ± std. deviation of triplicate measurements.

### MTT Cytotoxicity Assays

MDA-MB-231 cells were cultured in RPMI (Roswell Park Memorial Institute) media supplemented with 10% FBS (Fetal Bovine Serum), L-glutamine, and antibiotics. Cells (9 × 10^3^ cells/well) were seeded in 96-well plates. The next day, the cells were incubated with different concentrations of TOPI-4 (100 nM-40 μM) for 24 h, after which, MTT reagent (5 mg/mL) was added and the cells were incubated at 37°C for another 5 h. Then, the medium was slowly removed without breaking the formazan crystals. Next, 100 μL of SDS-DMF solution (80 g of SDS in 400 mL of 1:1 DMF:H_2_O) was added to stop the reduction reaction. Finally, the absorbance values at 570 nm were recorded, the data were analyzed using Graphpad Prism 7.0 and fitted to a sigmoidal dose response plot. The ED_50_ value of TOPI-4 was estimated to be 10.3 ± 0.1 μM and this concentration was used in further experiments to test the ability of β-CD to reverse the cell toxicity effect of TOPI-4.

## Results and Discussions

**Figure 1:**
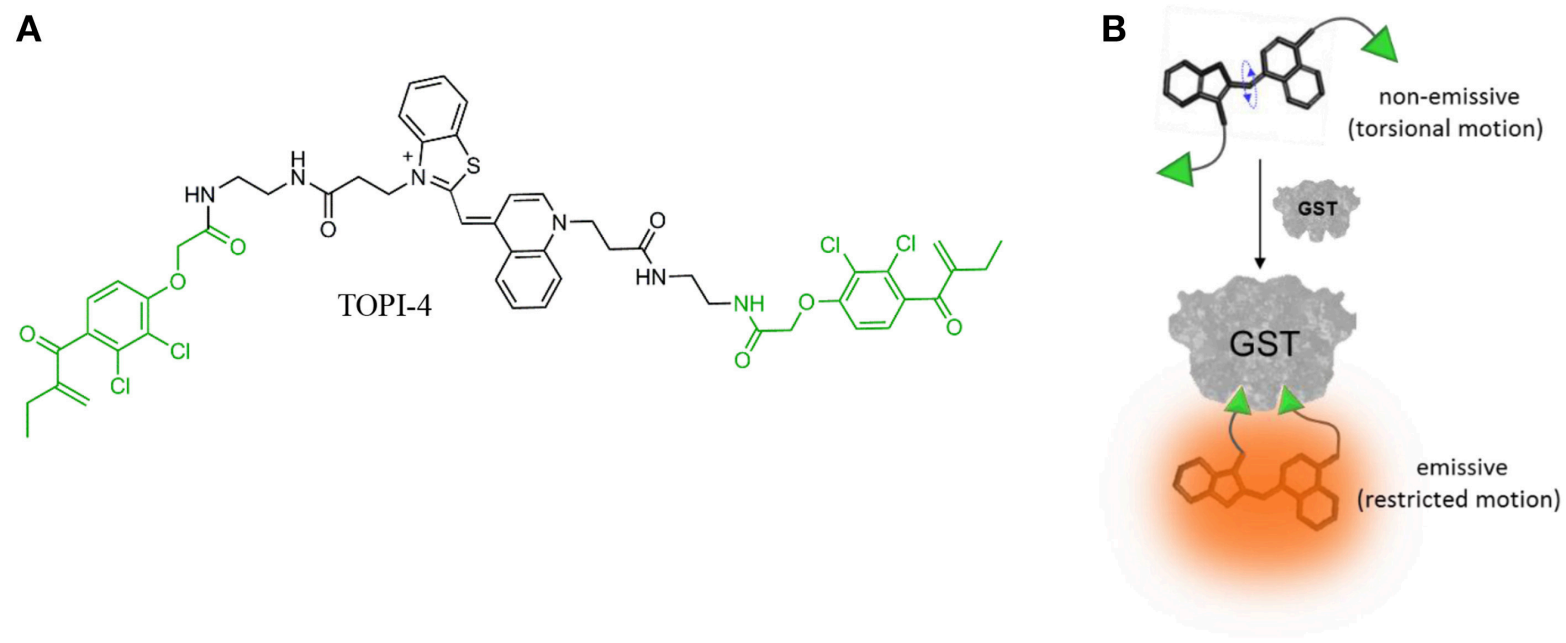
**(A)** Chemical structure of TOPI-4. The EA units are highlighted in green. **(B)** Schematic illustration showing how the binding of TOPI-4 to GST results in a “turn-on” emission signal.

**Figure 2:**
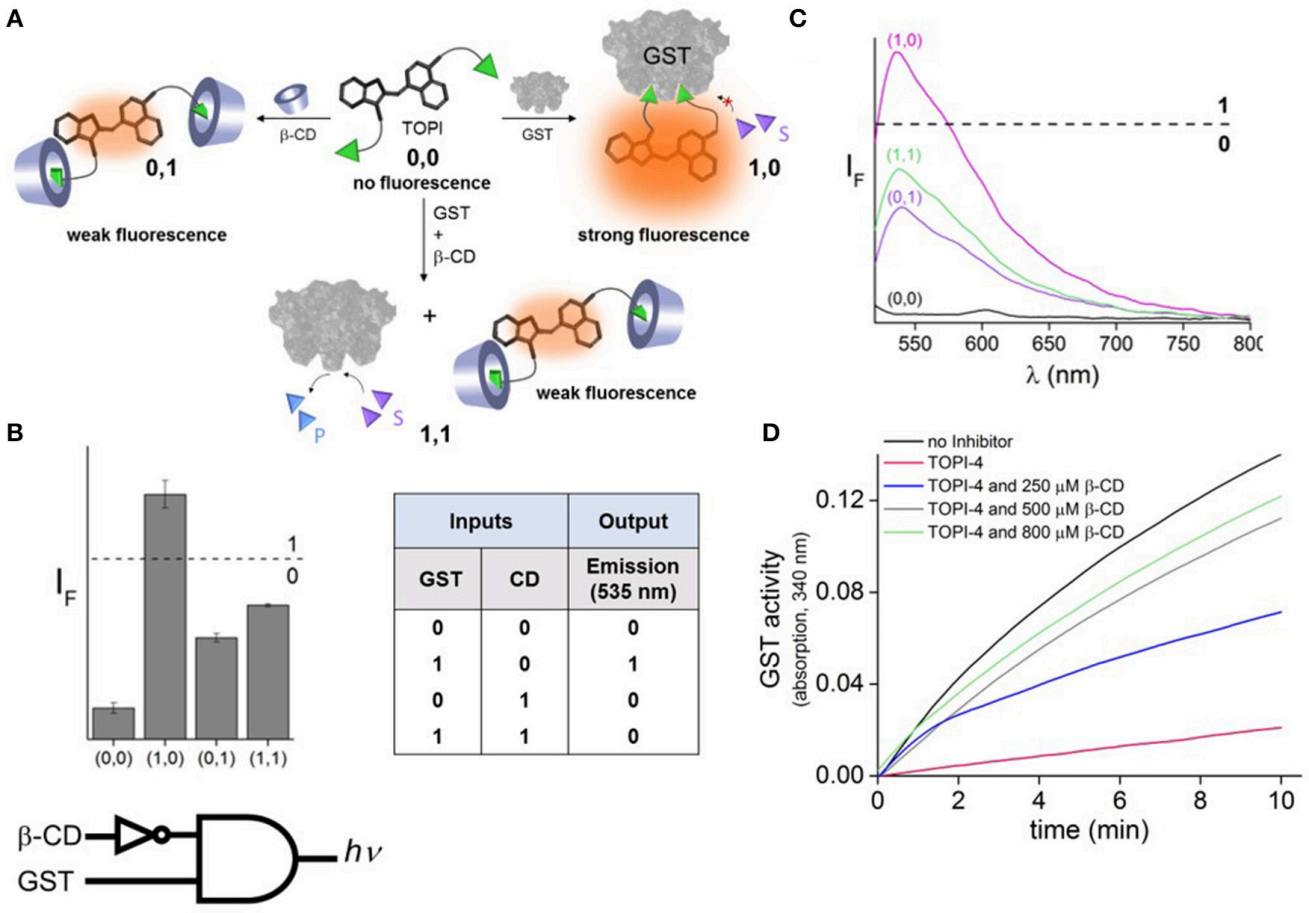
**(A)** Schematic illustration showing how the binding of TOPI-4 to GST and/or β-CD inputs affects the catalytic activity of GST and the emission of the probe, which results in an optical INHIBIT gate function. S and P correspond to the substrate and product, respectively. **(B)** The corresponding bar plot and truth table. The results are expressed as the mean values ± std. deviation of the triplicate measurements. **(C)** The fluorescence response of TOPI-4 (100 nM) to different combinations of the GST (90 nM) and/or β-CD (1 mM) inputs. **(D)** Enzymatic activity of GST (20 nM) in the absence and presence of TOPI-4 (500 nM) and with increasing concentrations of β-CD. λ_ex_/λ_em_ = 500/535 nm.

To demonstrate the feasibility of creating molecular logic gate-based chemotherapy antidotes, we exploited the inhibitory effect of bivalent, “turn-on” fluorescent molecular probes, termed TOPIs, recently developed by our group. In an earlier study, we showed that the binding of the two ethacrynic amide (EA) units of TOPI-4 (Figure 1A) to the two active sites of GST restricts the torsional motion of the probe, which leads to a “turn-on” emission signal (Figure 1B; Unger-Angel et al., 2015). This enhanced emission was selectively observed in biofluids and cancer cells with elevated levels of GSTs, indicating the potential of using such probes in medical diagnosis (Unger-Angel et al., 2015). GSTs are well-known targets for cancer therapy, and ethacrynic acid-based inhibitors have been considered as anti-cancer drug candidates (Allocati et al., 2018). This prompted us to investigate whether the fluorescent TOPI-4 probe could also act as a switchable GST inhibitor that, in addition to sensing GST and cancer cells (Unger-Angel et al., 2015), could be used to regulate GST activity and consequently, cancer cell death. In particular, we expected that analyzing the optical response of TOPI-4 to GST and an additional supramolecular host that interacts with TOPI-4 would indicate the host's ability to disrupt the GST-TOPI-4 binding interactions and the consequent inhibition of GST activity (Figure 2A). To prevent the inhibitory effect of TOPI-4 by a supramolecular “antidote,” we selected beta-cyclodextrin (β-CD) as the external input. β-CD was chosen as an input for two main reasons. First, this supramolecular host is known to form complexes with a variety of hydrophobic guests, about the size of the EA groups (Crini, 2014). Therefore, we expected that β-CD would form a complex with TOPIs, disrupt their binding to GST, and possibly limit their ability to penetrate cells. Second, β-CD is a non-toxic agent that has been tested for various medical applications (Bom et al., 2002; Del Valle, 2004; Loftsson et al., 2005). Hence, by using β-CD as an “antidote,” we aimed to demonstrate the relevance of this study to real-life applications. Figure 2A shows the way this additional β-CD input should convert TOPI-4 from a simple fluorescence switch (i.e., a YES gate), which previously responded to GST only (Unger-Angel et al., 2015), into a molecular INHIBIT gate whose fluorescence output depends on the combinations of GST and β-CD as inputs. Importantly, because the GST-TOPI-4 interaction should always be accompanied by enzyme inhibition, we expected that obtaining an optical INHIBIT function would indicate the ability of β-CD to serve as an “antidote” that prevents GST inhibition by TOPI-4 (Figure 2A, state 1, 1 vs. 1, 0). For simplicity, Figure 2A illustrates the formation of a 1:2 TOPI-4: β-CD complex. Note, however, that other modes of interaction that can prevent inhibition (e.g., binding of β-CD to the TO core) may also occur. The rationale behind the different optical states (Figure 2A) and the corresponding truth table (Figure 2B) is as follows: In the absence of the GST and β-CD inputs (state: 0, 0), TOPI-4 is not fluorescent (output: 0), owing to an intramolecular motion between the benzothiazole and the quinoline rings. The binding of TOPI-4 to GST (state: 1, 0), however, inhibits the enzyme and concomitantly restricts the intramolecular motion, which leads to a strong fluorescence response (output: 1). The interaction of TOPI-4 with β-CD alone (state: 0, 1) less significantly affects the intramolecular twisting of TOPI-4, which results in a weaker fluorescence signal (output: 0). Finally, when both GST and β-CD inputs are present (state: 1, 1), β-CD prevents the EA groups of TOPI-4 from binding to the two active sites of the GST dimer. Consequently, the probe remains in a low fluorescence state (outputs: 0) and GST remains active. The anticipated optical responses were measured experimentally (Figure 2C). As expected, the maximal fluorescence intensity obtained upon adding GST alone was not observed when other combinations of inputs were present in the solution. Importantly, the INHIBIT gate responses revealed similar fluorescence outputs for states (0, 1) and (1, 1), which indicates the possibility of using β-CD as an “antidote” that prevents the formation of the GST-TOPI-4 complex. Note that although this digital analysis of TOPI-4's optical responses provides an efficient analytical tool to distinguish among bimolecular interactions, the relatively small differences between the fluorescence intensity values generated for state (1, 1) (55%), state (1, 0) (100%), and (0, 1) (41%) would complicate using this INHIBIT gate to construct advanced molecular computation devices. For example, it would complicate integrating this gate with another gate that responds to light as an input. This research direction, however, is beyond the scope of this study.

The effect of β-CD on GST inhibition was confirmed by performing an enzymatic assay that follows the conjugation of two GST-specific substrates: chloro-2,4-dinitrobenzene (CDNB) and glutathione (GSH) (Figure 2D; Peri-Naor et al., 2015a). With this assay, the formation of the product (GSH-CDNB conjugate) was followed by monitoring the absorbance at 340 nm in the presence and absence of TOPI-4, as well as in the presence of TOPI-4 and increasing concentrations of β-CD. The results show that whereas 500 nM of TOPI-4 strongly inhibited the enzyme, in the presence of β-CD (800 μM) GST remained active, confirming that the supramolecular interactions between the β-CD host and the TOPI-4 guest disrupt the function of the TOPI-4 inhibitor.

**Figure 3:**
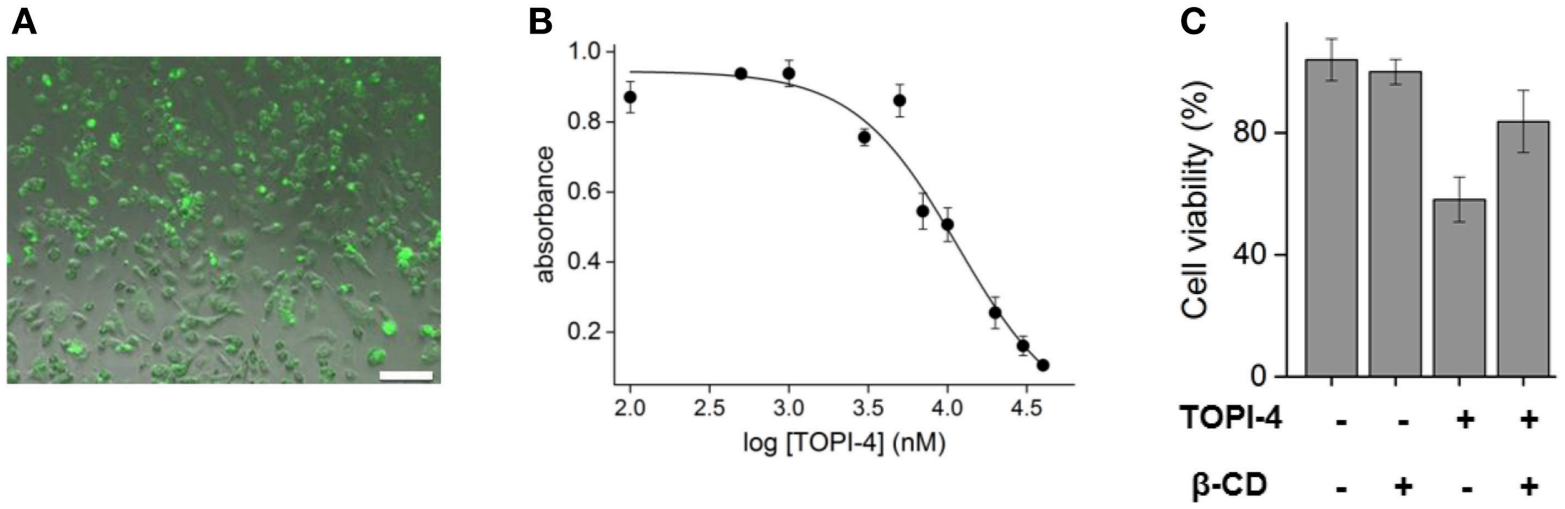
**(A)** Fluorescence image of MDA-MB-231, overexpressing GST, incubated with TOPI-4 (10 μM). The scale bar is 100 μm. **(B)** Results of an MTT assay that measures the viability of MDA-MB-231 cells under increasing concentrations of TOPI-4. The absorption at 570 nm reflects the number of viable cells. **(C)** Cell viability scores of MDA-MB-231 cells treated with TOPI-4 (10 μM) and/or β-CD (750 μM).

To demonstrate the relevance of these results to therapeutic applications, breast cancer cells (MDA-MB-231) overexpressing GST were treated with increasing doses of TOPI-4, imaged using a fluorescence microscope, and their viability was measured by using a MTT assay following ~24 h of incubations. The results show that TOPI-4 exhibits good cell permeability (Figure 3A) and that it inhibits cancer cell growth (Figure 3B) with an ED_50_ value of 10 μM. In the next step, we investigated whether, in addition to regenerating GST activity (Figure 2D), β-CD can serve as an “antidote” that reduces the TOPI-4-induced cell toxicity. Figure 3C shows the viability scores obtained upon subjecting breast cancer cells to 10 μM TOPI-4 with and without β-CD (750 μM). As a control, the number of viable cells was also measured in the presence of only β-CD. The results show that whereas TOPI-4 eradicated nearly 50% of the viable cells, in the presence of β-CD the cell viability increased to ~85%, indicating the potential of using supramolecular inputs of this class as “antidotes”. Notably, the β-CD “antidote” did not fully reverse the effect of the inhibitor on cells. Although this incomplete recovery of cell viability is consistent with the incomplete reactivation of GST in the enzymatic assay (Figure 2D), it could also result from non-specific interactions between the GST-TOPI-4 complex and other proteins, which leads to partial dissociation of the inhibitor-antidote complex in a cellular environment. Alternatively, it may indicate that, in addition to GST inhibition, other factors contribute to the TOPI-4-induced cytotoxicity.

**Figure 4:**
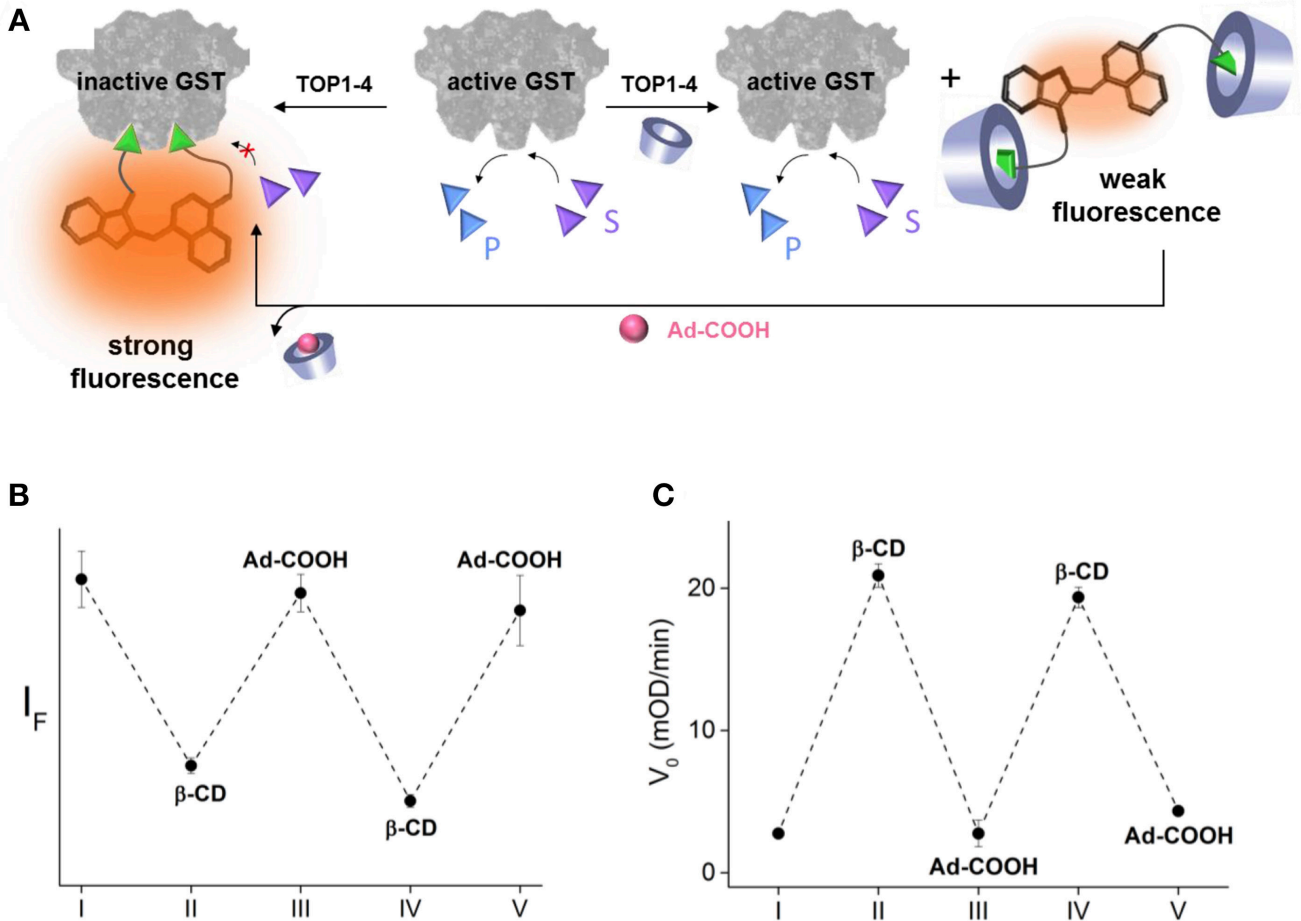
**(A)** Schematic representation of the way GST activity can be controlled by using Ad-COOH as an additional input that competes with the binding of β-CD to the TOPI-4 inhibitor. **(B)** Emission intensity measured by combining TOPI-4 with GST-P1-1 and different combinations of β-CD and Ad-COOH: (I) no input, (II) β-CD (1 mM), (III) β-CD (1 mM) and Ad-COOH (1.5 mM), (IV) β-CD (3 mM), and Ad-COOH (1.5 mM), and (V) β-CD (3 mM) and Ad-COOH (5 mM). **(C)** Initial velocity (V_0_) measured by combining TOPI-4 with GST-P1-1 and different combinations of β-CD and Ad-COOH: (I) no input, (II) β-CD (800 μM), (III) β-CD (800 μM), and Ad-COOH (1 mM), (IV) β-CD (2.5 mM) and Ad-COOH (1 mM), and (V) β-CD (2.5 mM) and Ad-COOH (3.3 mM). λ_ex_/λ_em_ = 500/535 nm.

In addition to demonstrating a model of a drug-antidote system, these results demonstrate a potential means to establish precise, temporal control over enzyme function. Inspired by the way that enzyme activities were reversibly controlled using DNA-based devices (Saghatelian et al., 2002; Harris et al., 2011; Peri-Naor et al., 2015a), we investigated the possibility of making our system reversible by introducing an adamantane (Ad) derivative as another chemical input. We expected that a water-soluble 1-adamantanecarboxylic acid (Ad-COOH) would compete with the binding of β-CD to the EA binders of TOPI-4, which would release the caged inhibitor and enable it to inhibit GST (Figure 4A). Ad-COOH was chosen for this goal, owing to the ability of Ad-derivatives to bind β-CD strongly and selectively (Wenz et al., 2006). In addition, Ad derivatives, similar to β-CDs, are generally non-toxic and have been tested as therapeutics (Štimac et al., 2017). The ability to obtain precise, supramolecular control over the binding of TOPI-4 to GST was demonstrated by adding GST to TOPI-4 incubated with increasing concentrations of β-CD and Ad-COOH and measuring the fluorescence responses (Figure 4B). The results show that the successive addition of an excess of β-CD and Ad-COOH led to a sequential decrease and increase in the fluorescence of TOPI-4, respectively, which is expected from the changes in the interaction between the fluorescent probe and the GST enzyme (Figure 4A). Next, we measured the initial velocity (V_0_) of the GST-catalyzed reaction measure after adding GST to a solution containing the CDNB and GSH substrates and increasing concentrations of β-CD and Ad-COOH (Figure 4C). Strikingly, whenever one of these inputs was introduced in excess, the effect of the other input was reversed, confirming the ability to control GST activity by using a supramolecular host-guest switching mechanism (Figure 4A). We believe that regulating intracellular enzymatic activities with similar mechanisms could also provide a means to establish precise, temporal control over cell growth. Research in this direction is underway in our laboratory.

## Conclusions

In conclusion, we have shown how the optical responses of a molecular logic gate revealed the possibility of using it to regulate enzyme function and consequently, cytotoxicity. Although much progress needs to be made in terms the inhibitor's affinity and selectivity in order to apply this system as therapeutics, this model system demonstrates a unique, supramolecular approach to achieving chemotherapy antidotes and switchable enzyme functions. Most importantly, it shows how concepts developed in the area of molecular logic gates, such as the use of supramolecular hosts as inputs that control the fluorescence responses of molecular logic gates (de Silva et al., 1999; Pischel et al., 2010), could be used to construct assays that track biomolecular interactions. Our results show the potential of using such assays for the generation of “smart” therapeutics, in which the function of enzymes and/or drugs can be controlled with external, non-toxic input signals. We hope that this study will inspire the development of other molecular computation devices that can operate in a complex biological environment, such as within living cells, and consequently, can perform tasks that are beyond the ability of conventional electronic computers.

## Author Contributions

LU-A, LM, and DM designed the project. LU-A synthesized the probe, performed the experiments, and analyzed the data. LM and DM wrote the manuscript.

### Conflict of Interest Statement

The authors declare that the research was conducted in the absence of any commercial or financial relationships that could be construed as a potential conflict of interest.
